# *In vivo* anti-ulcerogenic effect of okra (*Abelmoschus esculentus*) on ethanol-induced acute gastric mucosal lesions

**DOI:** 10.1080/13880209.2018.1442481

**Published:** 2018-03-07

**Authors:** Deniz Ortaç, Mustafa Cemek, Turan Karaca, Mehmet E. Büyükokuroğlu, Zafer Ö. Özdemir, Ayşe Tuba Kocaman, Sadık Göneş

**Affiliations:** a Department of Bioengineering, Biochemistry Division, Faculty of Chem. and Met. Eng., Yıldız Technical University, Istanbul, Turkey;; b Department of Histology and Embryology, Faculty of Medicine, University of Trakya, Edirne, Turkey;; c Department of Pharmacology, Faculty of Medicine, Sakarya University, Sakarya, Turkey;; d Technology Faculty, Kırklareli University, Kırklareli, Turkey

**Keywords:** Gastroprotection, apoptosis, gastric ulcer, immunohistochemistry, ulcer index, ulcer inhibition

## Abstract

**Context:** Okra, *Abelmoschus esculentus* (L.) (Malvaceae), is a medicinal plant widely used in Turkish traditional medicine for the treatment of various diseases such as ulcers and gastritis.

**Objective:** In the present study, we evaluated the gastroprotective effect of okra against ethanol-induced acute gastric mucosal injury in animal models.

**Materials and methods:** Wistar rats were treated with 500, 250 or 100 mg/kg okra; 20 mg/kg famotidine (Fam); and 75 mg/kg quercetin (Que). Following a 60 min period, all the rats were given 1 mL of ethanol (80%). One hour after the administration of ethanol, all groups were sacrificed.

**Results**: At 5000 mg/kg, the extract produced (okra) no signs of toxicity in animals. Okra 500, 250, 100, Fam 20 and Que 75 inhibited ulcer formation by 81.0, 67.5, 67.0, 76.3 and 72.4%, respectively. Okra 500 significantly decreased edema, hemorrhage and inflammation scores compared with the ethanol group (*p* < 0.05). The oxidant levels decreased significantly in the all study groups compared within ethanol group (*p* < 0.001). Serum β-carotene and retinol levels significantly increased 40.2 and 45.4% in the okra 500 group. In okra 500, 250 and Fam 20 groups, apoptosis significantly decreased (*p* < 0.001), while okra 500, 250 and Fam 20 groups showed a higher percentage of cell proliferation compared with the ethanol group (*p* < 0.001).

**Discussion and conclusions:** Our *in vivo* data indicate that okra has a gastroprotective effect against ethanol and could reduce the gastric ulcer as seen from biochemical and histopathological results. We suggest that okra could be a possible therapeutic antiulcer agent.

## Introduction

Due to the excessive usage of non-steroidal anti-inflammatory drugs, alcohol consumption or smoking, nutritional deficiencies, *Helicobacter pylori* infection and stressful lifestyle, gastric ulcer is still a major worldwide health problem. If ulcer is not properly treated, serious complications such as bleeding and perforation of stomach may occur. Proton pump inhibitors and multiple combinations of antibiotics are the most popular treatments despite their serious side effects and represent a high cost for the patient (Choi et al. 2009; Cemek et al. 2010; Jarret et al. 2011; Yesilada et al. 2014; Liju et al. 2015).

Ethanol is a commonly used as an ulcerogenic agent in humans and experimental animals and it causes damage of the gastric mucosa with severe gastric hemorrhagic lesions (Cemek et al. 2010; Liju et al. 2015). Thus, an experimental model of ethanol-induced gastric mucosal damage is often used. Ethanol is metabolized by alcohol dehydrogenases to acetaldehyde, is then further metabolized to acetic acid. Acetic acid has also toxic effects on the living tissues such as stomach. The gastric wall mucus secretion is considered to be an important defensive barrier that protects the gastrointestinal mucosa from toxins (Jarret et al. 2011; Liju et al. 2015; Sidahmed et al. 2015).

Medicinal plants are considered such as important sources of bioactive new molecules in the food. Numerous plants are used for medicinal purposes in many countries and traditional plant therapy methods for the treatment of many diseases are still popular among people. Therefore, several studies have been performed on the extraction of new anti-ulcer agents from natural sources by researchers (Choi et al. 2009; Cemek et al. 2010; Yesilada et al. 2014; Liju et al. 2015; Sidahmed et al. 2015).

Okra, *Abelmoschus esculentus* (syn. *Hibiscus esculentus*) (L.) (Malvaceae), an annual vegetable crop and known by many local names as lady's finger, bamya, gumbo, guino‐gombo, guibeiro and bhindi in different parts of the world. Okra has long been used in traditional medicine due to therapeutic potential in the Middle East, Far East and the Turkey (Shui and Peng 2004; Gemede et al. 2015; Xia et al. 2015). Okra as a valuable nutrient can be eaten as raw or cooked. Okra is rich in functional constituents such as amino acid, lipid, carbohydrate, dietary fiber, mineral and vitamin (A, C, E, K, β-carotene, folate, choline and niacin). Besides its nutritional content, it has beneficial components for health. Okra contains various polyphenolic and flavonoid components such as quercetin 3′-*O*-xylosyl glucoside, quercetin 3′-*O*-glucosyl glucoside, quercetin 3′-*O*-glucoside and quercetin 3′-*O*-(6″-*O*-malonyl)-glucoside, (−) epigallocatechin, rutin, hyperin, isoquercetin, hibifolin and myricetin (Ngoc et al. 2008; Jarret et al. 2011; Sabitha et al. 2011; Van Dam et al. 2013).

In traditional medicine, okra is believed to show diuretic, antispasmodic, demulcent and emollient poultice, cordial, laxative and anticancer effects (Ngoc et al. 2008; Van Dam et al. 2013). It is also known for its beneficial mucilage content. Furthermore, *in vivo* and *in vitro* antioxidant, antidiabetic antihyperlipidemic, antimicrobial, antifatigue, hepatoprotective and neuroprotective effects of okra have been reported (Sabitha et al. 2011; Van Dam et al. 2013; Gemede et al. 2015; Xia et al. 2015). Quercetin (Que) as an antioxidant bioactive molecule of okra has proven to exhibit free radical scavenging activity toward hydroxyl and peroxyl radicals and superoxides anions (Lengsfeld et al. 2004; Abourehab et al. 2015).

Famotidine (Fam) is the most potent H_2_-receptor antagonist in the clinical treatment of gastric diseases. It is primarily associated with effects on gastric acid secretion. Fam inhibits basal and stimulated gastric acid secretion in a dose-dependent manner after oral administration of 5–40 mg/kg (Abourehab et al. 2015). Although the beneficial effects of okra extract on human health have not yet been sufficiently examined on gastric mucosal injury in experimental studies. An *in vitro* study by Lengsfeld et al. (2004) examined extracts from okra on their ability to interact adhesively with *Helicobacter pylori* that is known to play a major role in the development of chronic gastritis. They found that okra has the antiadhesive effect *in vitro* and these antiadhesive qualities of okra were assumed to be due to a combination of glycoproteins and highly acidic sugar compounds. In another study, gastric mucus secretion levels were investigated in rats treated with *Abelmoschus esculentus* seed. According to this study, *Abelmoschus esculentus* increased the amount of gastric mucus secretion (Shrikant et al. 2011).

However, no paper was published on the potential anti-ulcer activity of okra on the gastric ulcer index, gastric ulcer inhibition, mucosal edema, hemorrhage, inflammation, epithelial erosion of mucosal tissue, cell proliferation and apoptosis. Therefore, in the present study, we evaluated the biochemical and histopathological anti-ulcerogenic effects of okra against ethanol-induced acute gastric mucosal injury in animal models.

## Materials and methods

### Chemicals

Sodium chloride, sodium dihydrogen phosphate, sodium hydroxide, metaphosphoric acid, sodium citrate, phosphoric acid, copper (II) sulfate, hydrazine sulfate, sodium acetate, perchloric acid, acetic acid, sulfuric acid, thiourea, hydrogen peroxide, reduced glutathione (GSH), thiobarbituric acid, phosphate buffer, butylated hydroxytoluene, trichloroacetic acid, EDTA, [5,5-dithiobis-(2-nitrobenzoic acid)], disodium hydrogen phosphate, phenyelendiamine, sodium azide, 2,4-dinitrophenylhydrazine, ethanol, acetonitrile, 2-propanol, hexane and Que were purchased from Sigma-Aldrich. Fam (Famodin^®^ 40 mg/kg Sandoz¸ Istanbul, Turkey) were used. All other chemicals and reagents used in this study were of analytical grade. Ultra-distilled water was used as solvent. Monoclonal anti-proliferating cell nuclear antigen (PCNA) antibody (MS-106-B, Thermo Lab Vision, Fremont, CA), Apoptosis detection kit (Apop Tag plus Peroxidase in situ Apoptosis Detection Kit S7101, Millipore, Billerica, MA), biotinylated secondary antibody (Ultra Vision Detection System-HRP kit, Thermo, Fremont, CA) and streptavidin peroxidase (Ultra Vision Detection System-HRP kit, Lab Vision, Fremont, CA) were also used.

### Plant material

The aerial parts of okra were collected in August 2014 from Amasya, Turkey (40.665180, 35.823376. The coordinates of the location). The plant was authenticated by Dr. H. Baykal, Department of Botany of Science Faculty, Rize University, Rize, Turkey. The fruits of okra were dried in the oven at 25 °C for three days. Air-dried okra was pulverized with a blender. This plant material (40 ± 2 g) was extracted in a Soxhlet apparatus using 95% ethanol (200 mL) for 96 h. Then, it was distilled with a rotary evaporator and stored at 4 °C and the hydroalcoholic extract obtained was used to treat the animals when needed.

### Experimental animals

Forty-nine male Wistar rats with a weight of 225–250 g were used for the experiment. The animal laboratory was without windows and contained automatic temperature (24 ± 2 °C) and lighting (12 h light/dark) controls. The rats were fed with standard laboratory chow and tap water before the experiment. Rats were divided into seven equal groups (*n* = 7) and housed in different cages. The investigation was conducted in accordance with the Guide for the Care and Use of Laboratory Animals published by the US National Institutes of Health (NIH Publication no. 85-23, revised 1996). The investigation was conducted in accordance with the World Medical Association Declaration of Helsinki, and approval has been received from Bağcılar Training and Research Hospital Experimental Research and Skills Development Centre in Istanbul, Turkey.

### Acute toxicity studies

The acute toxicity was determined on Swiss albino mice by the fixed dose method of OECD Guide line No. 420 given by CPCSEA. The animals were divided into six groups of seven animals and okra was administered by oral route. The six groups were divided to receive doses of 100, 250, 500, 1000, 3000 and 5000 mg of okra extract per kilogram of body weight. The mice were allowed food and water *ad libitum* and were kept under regular observation to determine their percentage of mortality and behavioral changes over a period of 24 h. Okra hydroalcoholic extract did not show any toxicity or death up to a dose of 5000 mg/kg.

### Gastric ulcer induced by ethanol/protective effect study

Anti-ulcerogenic effect of okra was investigated with the ethanol-induced ulcer model. The most effective the doses of okra extract, Fam and Que agents were chosen in previous studies (Carari et al. 2003; Shui and Peng 2004; Ngoc et al. 2008; Sabitha et al. 2011; Van Dam et al. 2013; Abourehab et al. 2015; Gemede et al. 2015; Xia et al. 2015). Twenty-four hours before the experiment, the rats were fasted giving them access only to water. On the day of the experiment, groups okra 500, okra 250 and okra 100 were administered with 500, 250 and 100 mg/kg okra, group Fam 20 was administered with 20 mg/kg famotidine, group Que 75 was administered with 75 mg/kg quercetin, group Ethanol was administered with 0.5 mL distilled water and group Sham (intact rats) was not administered with any agent. All of drugs were administered by gavage at the same volume (0.5 mL) and 60 min after drug treatment, a single dose of 1 mL ethanol (80%) was administrated to all of the animals except for Sham by gavage. One hour after the administration of ethanol, rats were sacrificed with 100 mg/kg dose of ketamine (Pfizer, Turkey), blood samples were taken by cardiac punctures, and stomachs were removed and opened along the greater curvature and washed in physiological saline solution.

### Quantification of ulcers

The total stomach area and the ulcerated stomach area were determined by a computer-imaging program. Ulcer index (UI) was determined using Equation (1):

(1) UI=Ulcerated stomach area/Total stomach area

The inhibition effect (%) of each protective material was calculated using Equation (2):

(2) Ulcer inhibition%=[UI in control-UI in test]×100/UIin control

### RP-HPLC analysis

The HPLC equipment consisted of a Shimadzu LC-20Avp system including two LC-20AD solvent delivery units, and SPD-20 A UV–vis photodiode array detector, and CBM-20 A system controller, a CTO-20 A column oven, a DGU-20A3 degaser, and injection valve with 20 µL loop. Data were processed using the Shimadzu Class VP 5.3 HPLC data system on a Pentium II 400 PC compatible computer. The column was an Inertsil ODS-4 (50 mm × 2.1 mm ×3 µm). The column temperature was 30 °C. Elution was performed using a mobile phase made up of pure water:acetonitrile:2-propanol (70:22:8) at a flow rate of 0.05 mL/min. Chromatograms were recorded at 370 nm. Que was identified by comparing retention times and UV–vis spectra, with an authentic compound (Carari et al. 2003).

### Biochemical analyses

Blood samples for biochemical analyzes were collected using heparinized/non-heparinized syringes, and placed into polystyrene micro-tubes. After clotting and centrifugation at 4000 *g* for 7 min, serum was removed using EDTA-treated Pasteur pipettes. Their blood cells that remained after the removal of plasma were washed with isotonic saline (0.89% NaCl), and the buffy coat was removed. The red blood cells were washed again with isotonic saline and further processed for the preparation of hemolysate. Serum, erythrocyte and tissue samples were immediately stored in polystyreneplastic tubes at −80 °C until the time of analysis.

Malondialdehyde (MDA) (as an important indicator of oxidative stress) levels were measured according to the method of Jain et al. (1989). The principle of this method is based on the spectrophotometric (Shimadzu, Japan) measurement of the color that occurs during the reaction of the thiobarbituric acid with MDA. Concentrations of thiobarbituric acid reactive substances were calculated by the absorbance coefficient of malondialdehyde–thiobarbituric acid complex and expressed in nmol/mL. Reduced glutathione (GSH) concentrations were also measured by a spectrophotometric method (Beutler et al. 1963). After lysing sample and removal of precipitate, disodium hydrogen phosphate and 5,5′-dithiobis-(2-nitrobenzoic acid) (DTNB) solution were added and the color formed at 412 nm was read.

Serum ascorbic acid (vitamin C) levels were determined after derivatization with 2,4-dinitrophenylhydrazine (Omaye et al. 1979). In brief, 1 mL of sample was made up to 2 mL with 10% TCA and mixed well. It was centrifuged at 3500 *g* for 10 min and 1 mL of the supernatant was mixed with 0.5 mL of 2,4-dinitrophenylhydrazine reagent (3 g of 2,4-dini-trophenylhydrazine, 4 g of thiourea and 50 mg of copper sulfate in 100 mL of 9 mol/L H_2_SO_4_) and incubated at 37 °C for 3 h. The tubes were removed and 2.5 mL of ice-cold 85% H_2_SO_4_ was added. It was mixed well and kept at room temperature for 30 min, and the absorbance was measured using a spectrophotometer at 520 nm. β-Carotene and retinol (vitamin A) concentrations were measured in the extracts of serum ethanol hexane (1:1:3, v/v) by spectrophotometry at 425 and 325 nm, respectively (Suzuki and Katoh 1990).

### TUNEL assay

The previously reported terminal deoxynucleotidyl transferase-mediated dUTP nick end labeling (TUNEL) method, which detects the fragmentation of DNA in the nucleus during apoptotic cell death *in situ* was employed using an apoptosis detection kit (Apop Tag plus Peroxidase *in situ* Apoptosis Detection Kit S7101, Millipore, Billerica, MA) (Gungor et al. 2014).

The number of PCNA and TUNEL positive cells in random high-power sections was counted by a light microscope (Olympus BX51, Japan) and incorporating a software analysis system (Version 2.11.5.1, Argenit Kameram, Istanbul, Turkey). In gastric mucosa, the numbers of PCNA and TUNEL positive cells in 15 different random (each rat) sections were counted, and the scores were converted to number of PCNA and TUNEL positive cells of per unit area (cell number/mm^2^).

### Immunohistochemical evaluations

The sections (6 µm) were incubated with a specific mouse monoclonal anti-proliferating cell nuclear antigen (PCNA) antibody (MS-106-B, ThermoLabVision, Fremont, CA), diluted 1:50 for 1 h at room temperature. Sections were washed three times with PBS and incubated with biotinylated secondary antibody (Ultra Vision Detection System-HRP kit, Thermo, Fremont, CA). Subsequently streptavidin peroxidase (Ultra Vision Detection System-HRP kit, Lab Vision, Fremont, CA) was added and sections were maintained at room temperature for 20 min. The chromogen 3-amino-9-ethyl-carbazole (AEC Substrate System, Thermo Scientific/Lab Vision) was used, and the sections were counterstained with haematoxylin (Wang et al. 2005).

### Histopathological evaluations

For histopathological evaluation, specimens of the gastric walls were fixed in 10% buffered formalin solution, and then embedded in paraffin. Paraffin sections were cut using the microtome at a thickness of 6 µm and stained with hematoxylin and eosin (H&E). The histopathological changes were evaluated by an experienced histological according to the method previously reported (a) mucosal edema (score 0–4: 0, no lesions; 1, weak edema; 2, mucosal weak edema; 3, mucosal plus submucosal weak edema and 4, mucosal plus submucosal strong edema), (b) hemorrhage (score 0–4: 0, no lesions; 1, hyperemia; 2, hemorrhagic spots; 3, large hemorrhagic areas and 4, common hemorrhagic areas), (c) inflammatory cell infiltration (score 0–3: 0, no infiltration; 1, superficial mucosal infiltration; 2, strong mucosal infiltration and 3, mucosal plus submucosal infiltration), and (d) epithelial cell loss (score 0–3: 0, no ulceration; 1, a few small ulcers; 2, large ulcers and 3, stomach full of ulcers with perforations). The evaluations were performed in the mucosal and submucosal layers (Laine and Weinstein 1988; Amirshahrokhi and Khalili 2015).

### Statistical analyses

Statistical analysis of the control group and the six experimental groups were compared using a one-way analysis of variance (ANOVA) and Tukey’s post test. A value of *p* < 0.05 was considered statistically significant. All values were expressed as mean standard deviation. Statistical tests were performed using SPSS version 12.0 PL for Windows.

## Results

### Anti-ulcer effect of okra on ethanol-induced gastric lesions

**Figure 1.**
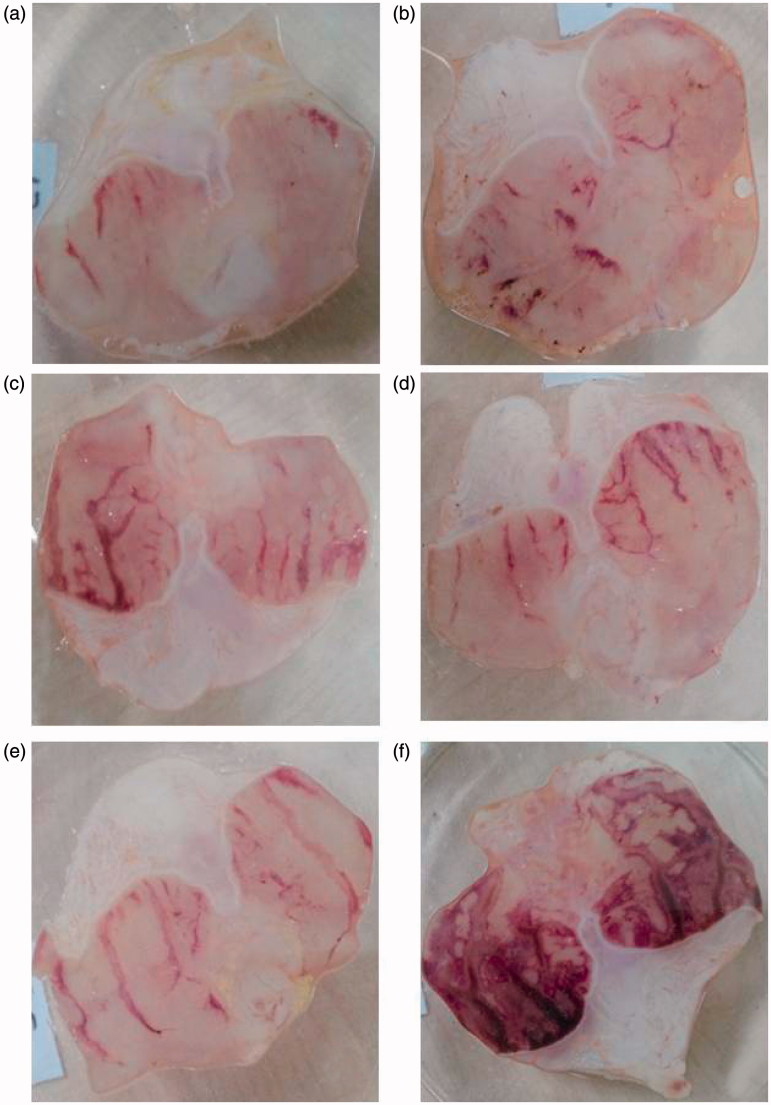
(a) Stomach of rat treated with okra 500; (b) Stomach of rat treated with okra 250; (c) Stomach of rat treated with okra 100; (d) Stomach of rat treated with Fam 20; (e) Stomach of rat treated with Que 75; and (f) Stomach of rat treated with ethanol (80%).

In Table 1 and Figure 1, the effect of orally administered okra on ethanol-induced gastric damage was shown as ulcer index and inhibition %.

**Table 1.** Ulcer index and the % ulcer inhibition levels in study groups.

	Ulcer index	Inhibition (%)
Okra 500	5.26 ± 2.4^a^	81.02
Okra 250	9.01 ± 3.9^a^	67.49
Okra 100	9.14 ± 3.5^a^	67.02
Fam 20	6.57 ± 2.7^a^	76.29
Que 75	7.65 ± 4.4^a^	72.40
Ethanol	27.72 ± 4.7	–
Sham	0	–

a Okra 500, Okra 250, Okra 100, Fam 20 and Que 75 with respect to Ethanol (*p* < 0.001).

One-way ANOVA and Tukey’s post test.

Okra 100, 250 and 500 mg/kg inhibited ulcer formation by 67.02, 67.49 and 81.02%, respectively. The dose of 500 mg/kg okra showed the highest inhibition, which was statistically (*p* < 0.05) different from the ethanol group (Table 1). Fam 20 mg/kg and Que 75 mg/kg, the positive controls, also offered inhibition of 76.29 and 72.40%, respectively, against ethanol induced gastric lesions and the effect was lower than that of okra 500 mg/kg.

### Biochemical findings

#### Effects on whole blood MDA and GSH levels

Table 2 presents the MDA and GSH levels in whole blood of experimental groups. The blood MDA level was minimum in okra 500 mg/kg group, and maximum in ethanol group. MDA levels decreased significantly (*p* < 0.001) in the okra 100, 250 and 500 mg/kg, Fam 20 mg/kg and Que 75 mg/kg treated groups compared within the ethanol group. The GSH levels, as endogenous agents that protect the body against oxidative stress, were found at the lowest level in the ethanol group. However, okra 250, 500 mg/kg and Fam 20 mg/kg treatment significantly inhibited the reduction in GSH levels, compared to ethanol group (*p* < 0.001, *p* < 0.05, respectively; Table 2).

**Table 2.** The levels (mean ± SD) of malondialdehyde (MDA) and reduced glutathione (GSH) in whole blood in study groups.

	MDA (nmol/mg whole blood)	GSH (nmol/g whole blood)
Okra 500	1.02 ± 0.2^a^	61.18 ± 3.79^a^
Okra 250	1.05 ± 0.2^a^	59.50 ± 3.1^a^
Okra 100	1.18 ± 0.2^a^	53.73 ± 3.7
Fam 20	1.29 ± 0.3^a^	57.34 ± 2.7^b^
Que 75	1.19 ± 0.2^a^	53.98 ± 3.9
Ethanol	2.56 ± 0.4	49.57 ± 4.5
Sham	0.97 ± 0.1^a^	91.43 ± 3.5^a^

a Okra 500, Okra 250, Okra 100, Fam 20, Que 75 and Sham with respect to Ethanol (*p* < 0.001).

b Fam 20 with respect to Ethanol (*p* < 0.05). One-way ANOVA and Tukey’s post test.

#### Effects on serum vitamin levels

Table 3 shows the antioxidant vitamin levels in the serum of experimental groups. No significant difference among serum ascorbic acid levels of experimental groups was observed. Only the group treated with 500 mg/kg dose of okra had a significant increase (*p* < 0.05) in β-carotene levels. Retinol levels significantly increased in okra 500 mg/kg group compared with those in ethanol group (*p* < 0.05).

**Table 3.** The levels (mean ± SD) of ascorbic acid, β-carotene and retinol in serum in study groups.

	Ascorbic acid (mg/dL)	β-carotene (µg/dL)	Retinol (µg/dL)
Okra 500	2.26 ± 0.2	19.12 ± 2.5^a^	49.17 ± 3.7^b^
Okra 250	2.18 ± 0.2	16.28 ± 3.1	44.08 ± 3.8
Okra 100	2.02 ± 0.2	14.86 ± 2.3	41.74 ± 3.3
Fam 20	2.08 ± 0.2	15.58 ± 2.8	42.06 ± 4.0
Que 75	1.94 ± 0.2	15.12 ± 2.5	41.90 ± 2.9
Ethanol	2.10 ± 0.3	13.63 ± 3.3	39.85 ± 3.5
Sham	2.29 ± 0.1	20.29 ± 2.4^b^	53.14 ± 2.5^c^

a Okra 500 with respect to Ethanol (*p* < 0.05). One-way ANOVA and Tukey’s post test.

b Okra 500 and Sham with respect to Ethanol *(p* < 0.01). One-way ANOVA and Tukey’s post test.

c Sham with respect to Ethanol (*p* < 0.001). One-way ANOVA and Tukey’s post test.

#### TUNEL findings

**Figure 2.**
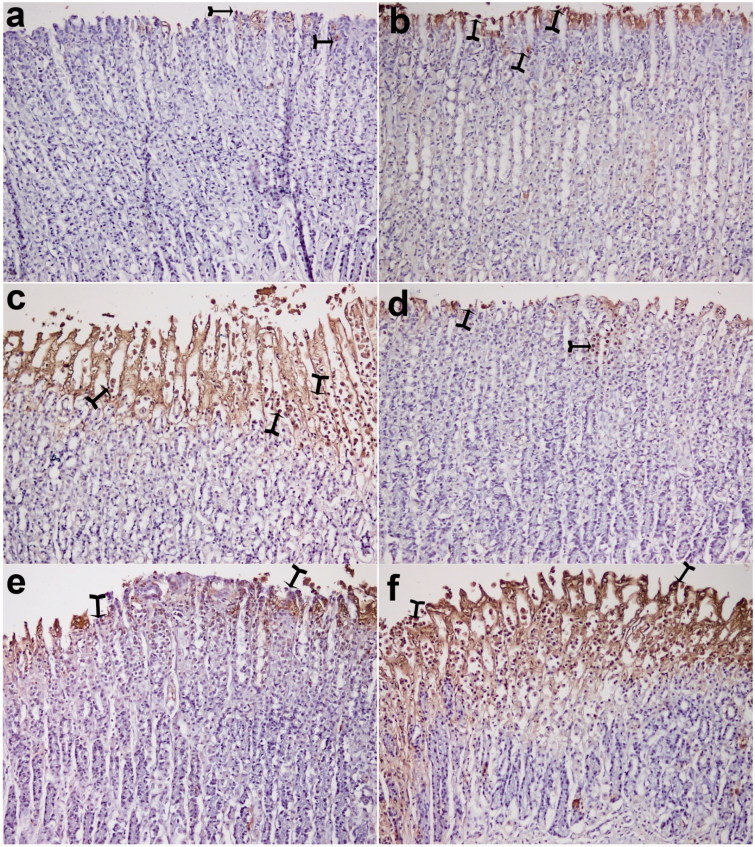
TUNEL assay. Nearly normal mucosa and minimal TUNEL positive cells were observed (a and b). TUNEL positive cells were observed in the intensive ethanol group (f), in the okra 100, Fam 20 and Que 75 and groups moderate amount of TUNEL-positive density was detected (c, d and e, respectively). (a) okra 500; (b) okra 250; (c) okra 100; (d) Fam 20; (e) Que 75 and (f) Ethanol groups. Arrows: TUNEL positive cells. Magnifications: 200×.

**Figure 3.**
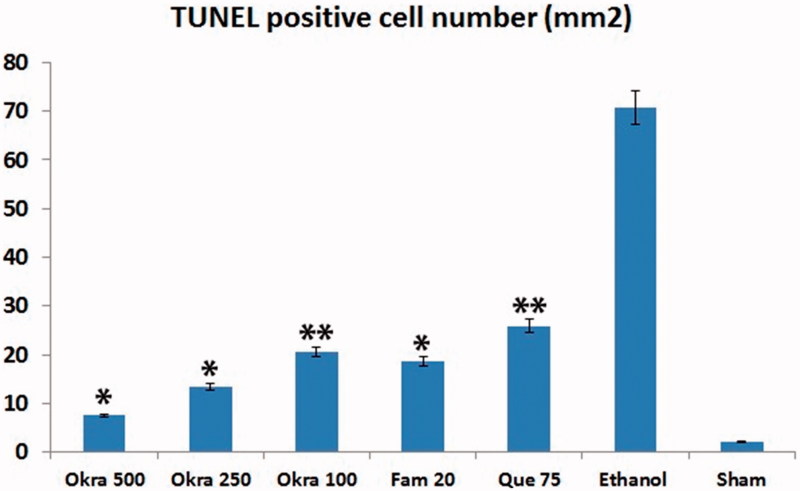
TUNEL positive cells distribution. TUNEL positive cell counts was higher in the ethanol group, but decreased significantly in the treatment group. *: *p* < 0.001; Compared with ethanol group; **: *p* < 0.05 Compared with ethanol group. One-way ANOVA and Tukey’s post test.

In the sham group, a few TUNEL-positive cells were observed in the gastric mucosa. The number of TUNEL-positive cells in the ethanol group was remarkably increased compared with the sham group. Treatment of okra and Fam significantly decreased the number of TUNEL-positive cells in the okra 500, 250 mg/kg and Fam 20 mg/kg groups compared with ethanol group (Figure 2). Besides, the number of TUNEL-positive cells in the okra 100 mg/kg and Que 75 mg/kg groups were lower than that of the ethanol group (Figure 3, *p* < 0.05).

#### Immunohistochemical analysis

**Figure 4.**
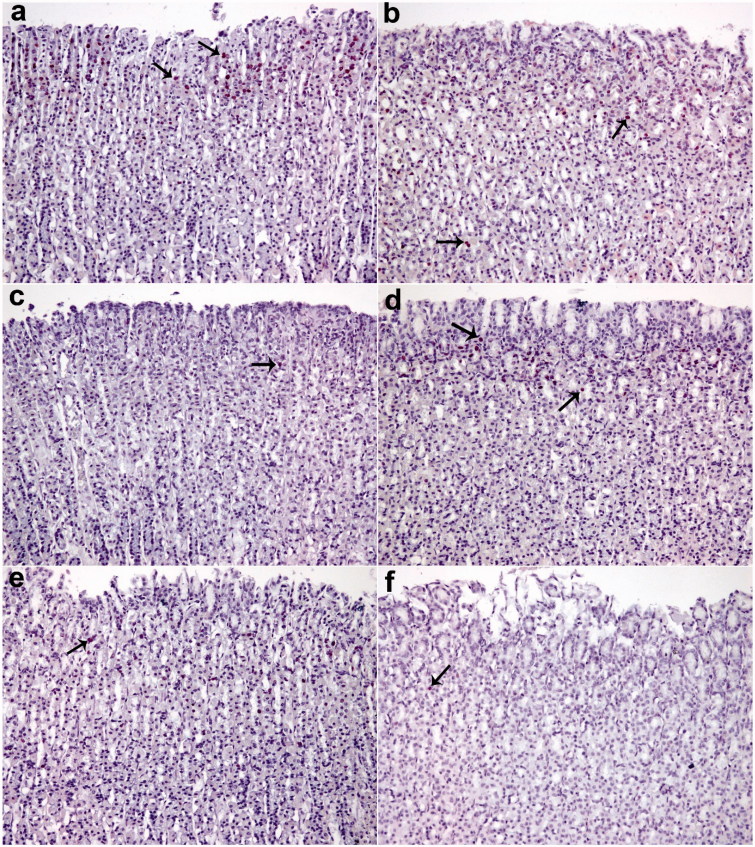
PCNA staining. The number of PCNA-positive cells in the intense okra 500 group (except sham); at least the group was determined in ethanol. (a) okra 500; (b) okra 250; (c) okra 100; (d) Fam 20; (e) Que 75 and (f) Ethanol groups. Arrows: PCNA positive cells. Magnifications: 200×.

**Figure 5.**
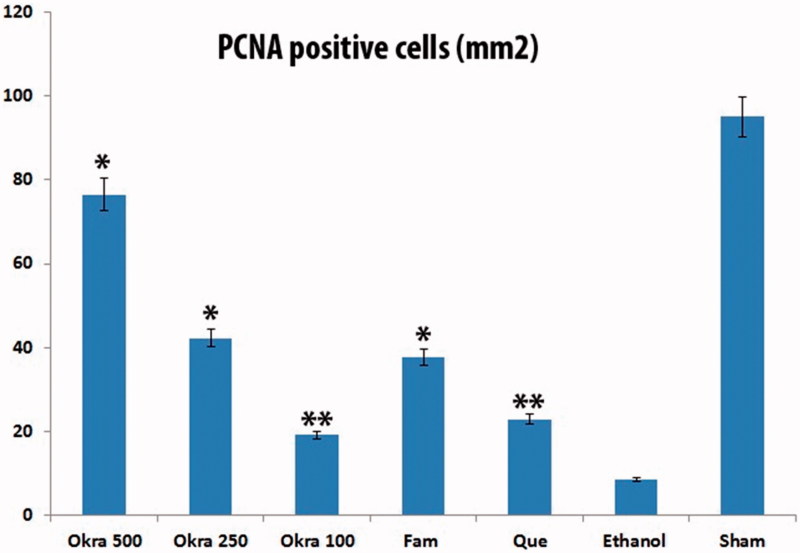
PCNA positive cells distribution. PCNA positive cell density less ethanol group; particularly in okra 500 group it was significantly increased in all treatment groups. *: *p* < 0.001; Compared with ethanol group; **: *p* < 0.05 Compared with ethanol group. One-way ANOVA and Tukey?s post test.

The gastric tissues obtained in the ethanol-induced gastric ulcer rat model were used for immunohistochemical localization of PCNA antibody. In the ethanol group, the absence of reactivity was observed due to the destruction of the epithelial layer, but it was likely that a rare/minimal activity in the layer adjacent to the ulcer was observed (Figure 4). In the rats treated with different doses of okra, Fam or Que, varying intense reactivities were observed with PCNA-positive nuclei marker. The okra 500 and 250 mg/kg groups showed a higher percentage of positive staining nuclei, suggesting that the treatment promotes an expressive increase in cell proliferation in the area of gastric mucosal healing (Figure 5).

### Histological evaluations

#### Evaluation of microscopic gastric damage

**Figure 6.**
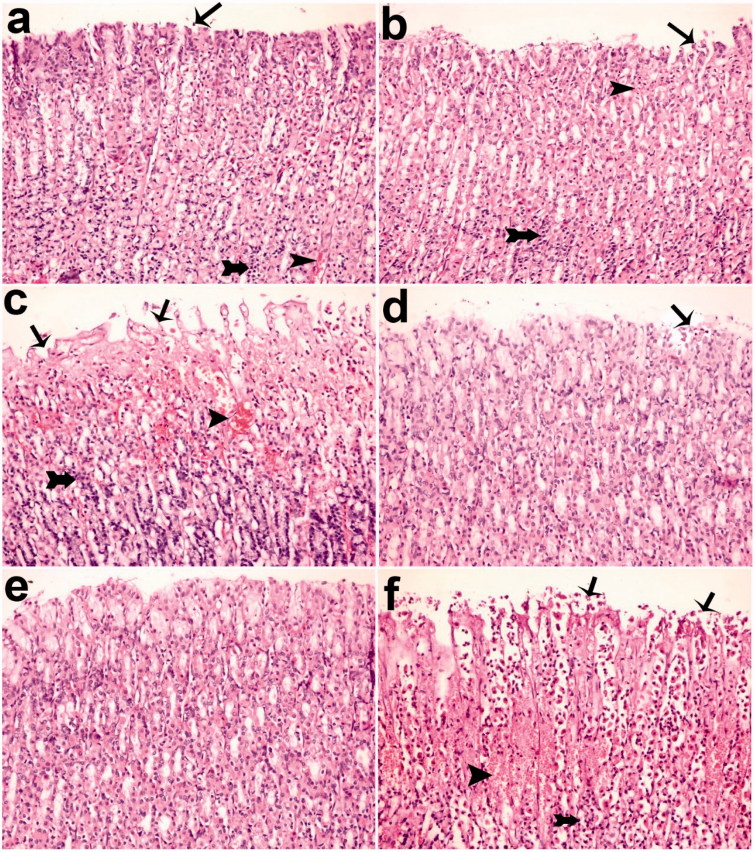
Histological appearance of gastric mucosa. Nearly normal mucosa view, okra 500 (a) and okra 250 (b); Hemorrhage, epithelial ulceration and very edema okra 100 (c); Decreases ulceration, hemorrhage and inflammation (d) Fam 20 and (e) Que 75 groups; Very epithelial ulceration, hemorrhage, inflammation and edema (f) Ethanol group. (H&E; Magnifications: 200×). Thin Arrows: epithelial ulceration; arrow-heads: Hemorrhage; Thick arrows: Inflammatory cells.

After the common gastric mucosal damage was induced by ethanol, histological examination of the gastric mucosa was characterized by detecting hemorrhagic, mucosal/submucosal edema, inflammatory cell infiltration and mucosal epithelial cell loss (Table 4; Figure 6). As shown in Table 4 and Figure 6, okra at a dose of 500 mg/kg significantly decreased edema, hemorrhage and inflammation scores compared with ethanol groups (*p* < 0.05). The effect of okra 500 mg/kg on hemorrhage was not statistically significant compared with the okra 250 mg/kg, Fam 20 mg/kg and Que 75 mg/kg groups.

**Table 4.** The effect levels of Okra 100, 250, 500 mg/kg, Fam 20 mg/kg and Que 75 mg/kg in gastric mucosa induce by ethanol in rats.

	Edema	Hemorrhage	Cell infiltration	Epithelial cell loss
Okra 500	0.7 ± 0.5^a^	0.5 ± 0.2^a^	0.7 ± 0.3^a^	0.7 ± 0.1^a^
Okra 250	0.8 ± 0.07^a^	1.0 ± 0.3^a^	1.1 ± 0.2	0.9 ± 0.7^a^
Okra 100	2.3 ± 1.0	2.1 ± 0.5	1.3 ± 0.4	2.3 ± 0.4
Fam 20	0.9 ± 0.2^a^	0.5 ± 0.1^a^	0.2 ± 0.1^a^	1.0 ± 0.7^a^
Que 75	1.0 ± 0.5^a^	0.8 ± 0.4^a^	0.6 ± 0.2^a^	1.3 ± 0.5^a^
Ethanol	2.5 ± 0.8	2.2 ± 0.2	1.7 ± 0.3	2.9 ± 0.7
Sham	0	0	0	0

a Okra 500, Okra 250, Fam 20, Que 75 with respect to Ethanol (*p* < 0.05). One-way ANOVA and Tukey’s post test.

#### RP-HPLC analysis

**Figure 7.**
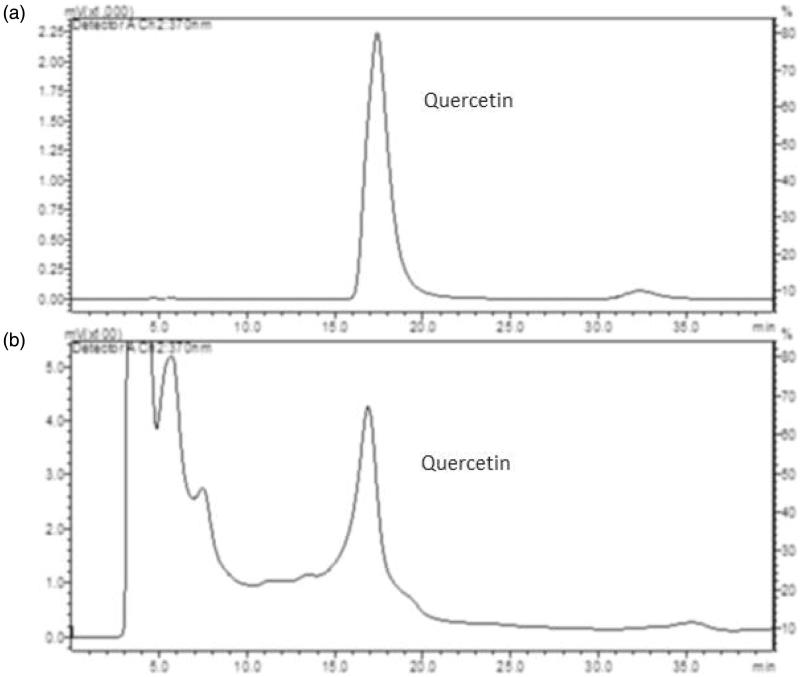
(a) HPLC choromatographic separation of quercetin standard. (b) Separation of quercetin in okra hydroalcoholic extract. For chromatographic conditions, see experimental procedures.

Using the HPLC method, pure standard quercetin and okra hydroalcoholic extract peaks were resolved under isocratic elution with retention times of 17.40 ± 0.33 and 16.881 ± 0.35 min, respectively, as illustrated in Figure 7.

## Discussion

Various medicinal plants such as okra have attracted growing attention due to their probable therapeutic benefits in most areas of the world (Jarret et al. 2011; Yesilada et al. 2014; Liju et al. 2015; Sidahmed et al. 2015). In this study, the antiulcer effect of okra was investigated in rats, using an ethanol-induced ulcer model. Therefore, the effect of okra on oxidant and non-enzymatic antioxidants and also, ulcer index, % ulcer inhibition, mucosal edema, hemorrhage, inflammation, epithelial erosion of mucosal tissue, cell proliferation and apoptosis were evaluated. Experimental results of the present study showed that okra considerably decreased the ethanol-induced ulcer lesions at all doses used (Tables 1 and 4; Figures 1 and 6). Immunohistochemical analysis in the present study indicates that TUNEL positive apoptotic cell quantity significantly decreases and the PCNA cells increase in treatment with okra 500, 250 mg/kg doses. The biochemical, histopathological, microscopic and macroscopic results show that okra treatment protects against gastric ulcers (Figures 1–6). The gastroprotective effect of okra is probably due to the presence of polyphenolic and flavonoid components such as quercetin (Que) (Shui and Peng 2004; Gemede et al. 2015; Xia et al. 2015).

The gastroprotective activity of quercetin has been reported in different animal studies and most investigators have focused on its possible multiple mechanisms (Sabitha et al. 2011; Van Dam et al. 2013; Gemede et al. 2015; Xia et al. 2015). Ertuğ et al. (2013) showed that Que reduce oxidant level and enhanced total antioxidant level in the presence of high superoxide anions, and Que protects NO from endogenous superoxide anion-driven inactivation and enhances its biological activity. In another study, reported that Que protects DNA damage induced by *H. pylori*-infected gastric mucosa cells (Klipinska et al. 2006). Additionally, it has been confirmed that the Que treatment significantly inhibits the ethanol-induced gastric lesions (Abourehab et al. 2015). Also, Ekström et al. (2011) showed that Que intake was strongly negatively associated with the risk of gastric cancer. The major components of okra are polysaccharide, amino acid, lipid, mineral and vitamin (Van Dam et al. 2013; Gemede et al. 2015; Xia et al. 2015). The major components in okra probably may affect the gastrointestinal mucosa regeneration or may be a protective layer against ethanol-induced gastric ulcer. In an experimental study showed that Que derivative induces mucus secretion and down-regulates ICAM-1 expression in protecting gastric damage induced by indomethacin (Yan et al. 2011). Shrikant et al. (2011) found that pretreatment with the paste of *A. esculentus* significantly increased the amount of gastric mucus content. Okra have strong antioxidant properties through free radicals scavenging such as superoxide anion, hydroxyl radical and nitric oxide and also, have strong effects of gastrointestinal mucosa regeneration. Okra may have shown gastroprotective activity with synergic effects.

Ethanol-induced gastric mucosal damage is an important experimental model usually utilized for preclinical evaluation of agents with potential anti-ulcer activity since ethanol has been regarded as a leading cause of gastric ulcer in humans (Salga et al. 2012). It is characterized by pathophysiological changes, such as hemorrhage, edema, inflammatory infiltration and epithelial cell loss (Cemek et al. 2010; Jarret et al. 2011; Liju et al. 2015; Sidahmed et al. 2015). The results of the present study indicate that a single dose of ethanol caused acute gastric bleeding and hemorrhagic lesions in the rat stomach (Figure 1). Pretreatment with okra prevented histopathological changes in the form of hemorrhage, edema, necrosis, inflammatory and dysplastic changes, erosions and ulceration in the gastric tissue (Figures 1–6). Apoptosis, programmed cell death, is an important mechanism maintaining homeostasis during growth, and it is a response to external stimuli in animal and human organisms. Arab et al. (2015) suggested that ethanol-induced ulcer increases apoptosis in gastric mucosa by raising caspase-3 activity. In the present study, a number of apoptotic cells significantly decreased in the animals treated with 500, 250 mg/kg of okra (Figure 2 and 3). The decrease in gastric mucosal apoptosis can be ascribed to the observed suppression of lipid peroxidation and TNF-α since excessive exposure of gastric mucosa to reactive oxygen species (ROS) and TNF-α has been reported to enhance gastric epithelial apoptosis (Liju et al. 2015; Sidahmed et al. 2015; Xia et al. 2015). Previous studies evidenced that ROS are implicated in the pathogenesis of ethanol-induced gastric mucosal injury (Cemek et al. 2010; Liju et al. 2015; Sidahmed et al. 2015). ROS are unpaired molecules generated as normal products during the mitochondrial respiration and from the peroxisomes to catalyze different redox reactions within the living organisms (Uner et al. 2010; Caksen et al. 2014) When ROS production accumulates and exceeds over the cellular antioxidant system, oxidative stress occurs (Aslan et al. 2012) Lipid peroxidation, an outcome of ROS reaction against cell membrane, generates extreme lipid peroxidation products such as MDA, causing oxidative gastric damage. MDA is used to determine lipid peroxidation levels. It is suggested a relationship between gastric ulcer and increased MDA levels and reported that MDA production has been enhanced following ethanol-induced gastric tissue injury (Birdane et al. 2007; Cemek et al. 2010). In an experimental study on the gastroprotective effect of *M*. *pruriens*, it was demonstrated that *M*. *pruriens* increased the antioxidant production, and decreased the MDA level in the respective pretreated groups (Liju et al. 2015). In this present study, all doses of okra significantly decreased the MDA levels (*p* < 0.001) (Table 2). This finding emphasizes the ability of okra to increase the antioxidant level, free radical scavenging activity, polysaccharide content and decrease the lipid peroxidation.

GSH is a tripeptide, an important non-enzymatic cytosolic antioxidant, and acts as a reductant/cofactor for some antioxidant enzymes (Cemek et al. 2006; Ertuğ et al. 2013). A previous study reported the possibility of zerumbonein raising the complementary action of gastric mucosal protective factors, particularly a significant increase in the endogenous antioxidant GSH was accompanied by the reduction of lipid peroxidation level (Shui and Peng 2004). Our study showed that all treatments with Fam 20 mg/kg, Que 75 mg/kg and okra 100, 250, 500 mg/kg increased the GSH content. Fam is a commercial drug used in the treatment of gastric ulcer. Our previous study revealed that Fam played a significant role in the inhibition of ethanol-induced ulcer (Birdane et al. 2007; Cemek et al. 2010). On the other hand, the treatment with a combination of Fam and Que considerably hindered the decrease of GSH levels, and the gastric tissue regained its normal GSH levels after treatment (Abourehab et al. 2015). In our study, a meaningful increase in GSH levels was observed with okra 500 mg/kg treatments, being comparable with Fam 20 mg/kg treatment (Table 2). Our results showed that the gastroprotective effect of okra 500 mg/kg was higher than the Fam 20 mg/kg.

The non-enzymatic antioxidants such as ascorbic acid, retinol and β-carotene play an important acute and chronic role in reducing or eliminating oxidative damage produced by ROS. The non-enzymatic antioxidants exert an antioxidant action by itself (scavenges ROS) and, thus, protect cellular membranes against oxidative stress initiated by several factors (Winkler et al. 1994; Ertuğ et al. 2013; Caksen et al. 2014). Retinol plays an important antioxidant role in biological systems, as well. The current study revealed that there was no significant difference between the groups in ascorbic acid levels. The serum β-carotene and retinol concentrations of the okra 500 mg/kg group rats were significantly higher than the ethanol group rats (Table 3).

The present study showed that the number of proliferating cells in the stomachs treated with okra 500 and 250 mg/kg doses was much higher than the ethanol group via PCNA immunohistochemical analysis (Figures 4 and 5). As a result, the increase in the number of PCNA positive cells showed that okra has a healing effect on ethanol-induced-gastric ulcer model.

The toxicity symptom of the okra was not recorded in mice. Also, LD_50_ value was not observed up to 5000 mg/kg of the okra extract in the present study. The mice did not show any abnormal signs and diarrhea in the acute toxicity study. Okra is a therapeutic vegetable that could be safely consumed.

## Conclusions

The biochemical and histopathological results of the present study indicate that okra has potent gastroprotective effects against ethanol-induced gastric ulcer. Okra reduced the gastric injuries, lipid peroxidation, ulcerated area, edema, hemorrhage, cell infiltration, epithelial cell loss and improved antioxidant defense systems. We suggest that okra may be a useful therapeutic antiulcer agent.
